# HDL-C and non-HDL-C levels are associated with anthropometric and biochemical parameters

**DOI:** 10.1590/1677-5449.180109

**Published:** 2019-04-01

**Authors:** Sandra Maria Barbalho, Ricardo José Tofano, Marcela Bueno de Oliveira, Karina Rodrigues Quesada, Mariana Ricci Barion, Marina Cristina Akuri, Marie Oshiiwa, Marcelo Dib Bechara

**Affiliations:** 1 Universidade de Marília – UNIMAR, Faculdade de Medicina, Departamento de Bioquímica e Farmacologia, Marília, SP, Brasil.; 2 Faculdade de Tecnologia – Fatec Marília, Departamento de Bioquímica e Nutrição, Marília, SP, Brasil.

**Keywords:** non-HDL-c, glycemia, dyslipidemia, metabolic syndrome, não HDL-c, glicemia, dislipidemia, síndrome metabólica

## Abstract

**Background:**

Dyslipidemias are associated with atherosclerosis and cardiovascular diseases. Recently, non-high-density lipoprotein cholesterol (non-HDL-c) has emerged as a new target for assessment and prediction of risk of cardiovascular disease (CVD) and is closely associated with atheroma plaque progression.

**Objectives:**

To evaluate associations between HDL-c and non-HDL-c levels and anthropometric and biochemical parameters and with the Castelli risk indexes I and II.

**Methods:**

300 randomly selected people were subdivided into two groups: patients with normal values for non-HDL-c and patients with altered values for non-HDL-c. These parameters were analyzed for associations with glycemia, total cholesterol (TC), triglycerides (TG), low-density lipoprotein (LDL-c), Castelli Index I (CI-I), Castelli Index II (CI-II), waist circumference (WC), body mass index (BMI) and presence of metabolic syndrome (MS).

**Results:**

Glycemia, TC, TG, LDL-c, CI-I, CI-II, WC and BMI were all significantly different between subjects with normal and altered values of HDL-c and non-HDL-c. TC and WC both exhibited significantly higher values among patients with abnormal non-HDL-c when compared to patients with abnormal HDL-c. A significant difference was observed in occurrence of MS among patients with altered values of HDL-c and non-HDL-c.

**Conclusions:**

Our results show that both HDL-c and non-HDL-c are associated with insulin resistance, dyslipidemia, atherogenic indices, and obesity. There is therefore a need for randomized clinical intervention trials examining the potential role of non-HDL-c as a possible primary therapeutic target.

## Resumo

**Contexto:**

A dislipidemia está associada à aterosclerose e às doenças cardiovasculares. Recentemente, a lipoproteína de não alta-densidade de colesterol (não HDL-c) emergiu como um novo alvo para avaliação da predição de risco de doença cardiovascular, intimamente associada à progressão da placa de ateroma.

**Objetivos:**

Avaliar as associações de níveis de HDL-c e não HDL-c com parâmetros antropométricos e bioquímicos, bem como com índices de Castelli I e II (CI-I e CI-II).

**Métodos:**

Trezentas pessoas selecionadas aleatoriamente foram divididas em dois grupos: pacientes com valores normais de não HDL-c e pacientes com valores alterados de não HDL-c. Esses parâmetros foram associados a glicemia, colesterol total (CT), triglicerídeos (TG), lipoproteína de baixa densidade (LDL-c), CI-I, CI-II, circunferência de cintura (CC), índice de massa corporal (IMC) e presença de síndrome metabólica (SM).

**Resultados:**

Glicemia, CT, TG, LDL-c, CI-I, CI-II, CC e IMC exibiram valores significativamente maiores para o não HDL-c quando comparado ao HDL-c. Uma diferença significativa na ocorrência de SM foi encontrada em pacientes com valores alterados de HDL-c e não HDL-c.

**Conclusões:**

Nossos resultados mostram que tanto o HDL-c quanto o não HDL-c estão associados a resistência à insulina, dislipidemia, índices de aterogênese e obesidade. Assim, há uma necessidade de futuros ensaios randomizados de intervenção clínica examinando o papel potencial do não HDL-c como possível alvo terapêutico primário.

## INTRODUCTION

 Dyslipidemias are major risk factors for development of atherosclerosis and cardiovascular diseases (CVD). They are associated with formation of atherosclerotic plaques, which build up in the intima of the vessel and can influence increased risk for cardiovascular events. Many risk factors play fundamental roles in dyslipidemias, including smoking, obesity, physical inactivity, high blood pressure, insulin resistance, and environmental and genetic influences. 1 ^-^ 5

 CVD is strongly associated with the Metabolic Syndrome (MS), which comprises a series of risk factors associated with high morbidity. The etiology of MS involves genetic, metabolic and environmental factors and it usually manifests with insulin resistance and abnormal values of HDL-c, triglycerides (TG), waist circumference (WC), blood pressure, and C-reactive protein (CRP), in addition to obesity. 1

 Atherosclerosis is associated with hypertriglyceridemia, low HDL-c levels, qualitative changes in LDL particles, accumulation of remnant lipoproteins, and postprandial hyperlipidemia. 1 ^,^ 2

 Recently, non-High-Density Lipoprotein cholesterol (non-HDL-c) has emerged as a new target for the prevention of cardiovascular events. It reflects the full burden of the cholesterol transported in atherogenic lipoproteins, it is crucial to prediction of CVD risk, 3 and it is closely associated with plaque progression. 4 ^,^ 5

 Non-HDL-c comprises the cholesterol carried by all potentially atherogenic particles, including LDL-c, intermediate density lipoproteins, very low-density lipoproteins, and remnant lipoproteins. Several meta-analyses found that non-HDL-c correlated more closely with cardiovascular risk than LDL-c, both at baseline and during therapy. 6 ^-^ 8

 Routes to earlier detection of CVD such as high sensitivity C-reactive protein, carotid intima-media thickness, and coronary artery calcium are among the various measures that can be used to screen for CVD in asymptomatic populations. 5 The atherosclerosis guidelines of the Brazilian Cardiology society, in common with other global guidelines, indicate that non-HDL-c is relevant to prevention and is a therapeutic target for management of CVD. 6 ^-^ 8

 This study aimed to evaluate associations between HDL-c and non-HDL-c levels and anthropometric and biochemical parameters in a group of patients. 

## METHODS

 The study population comprised 300 people who were included in our study after signing consent forms (in accordance with the Helsinki Declaration). 

 Participants were divided into two groups: patients with normal HDL-c and non-HDL-c levels and patients whose test results were abnormal. 

 All participants underwent a physical examination, including measurement of height, weight, and waist circumference (WC). Body mass index (BMI) was evaluated as weight in kilograms divided by height in square meters. 1 The anthropomorphic measurements weight, height, and WC were obtained using techniques recommended by Gibson. 2 For evaluation of anthropometric parameters, we used a digital scale (FILIZOLA® with capacity for 150 kg) and a fixed stadiometer with a metric scale, positioned on a flat surface. 1

 Glycemia, total cholesterol (TC), triglycerides (TG), and low-density lipoprotein (LDL-c) levels, waist circumference (WC), body mass index (BMI), and presence/absence of metabolic syndrome (MS) were also evaluated. The Castelli Risk Index I (CI-I) and Castelli Risk Index II (CI-II) were also considered. These two evaluations are derived from TC/HDL-c and LDL-c/HDL-c respectively. Values of HDL-c lower than 40mg/dL for men and 50mg/dL for women were considered altered. 

 Student's *t* test was used to compare parametric data and the Mann Whitney test was used for nonparametric data, with a significance level of 5% (p <0.05). 

## RESULTS

Table 1 lists the biochemical and anthropometric parameters for the patients, grouped by normal or abnormal HDL-c and non-HDL-c levels. Glycemia, TC, TG, LDL-c, CI-I, CI-II, WC, and BMI were all significantly different between patients with normal and altered HDL-c and non-HDL-c levels. TC and WC both exhibited significantly higher values among patients with abnormal non-HDL-c when compared to patients with abnormal HDL-c. 

**Table 1:** Biochemical parameters according to classification by HDL-c and non-HDL-c.

**Parameters**	**HDL-c**		**Non-HDL-c**	
	**Normal**	**Altered**	**Normal**	**Altered**
Glycemia	109.3±26.2 A	133.6±14.43 B	111.0±21.9 A	134.2±13.8 B
TC	188.9±31.1BC ^*^	186.8±42.34B	143.0±21.73 A	207.7±31.0 C
TG	112.7±21.4 A	147.5±24.9B	115.6±51.4 A	158.4±91.5 B
LDL-c	97.6±34.1 A	121.9±24.2 B	74.5±20.0 A	127.8±41.2 B
ICI	2.8±0.6 A	4.8±2.1 B	3.3±0.8 A	5.2±2.2 B
ICII	1.5±0.6 A	2.9±2.0 B	1.7±0.6 A	3.3±2.1 B
WC	91.0±14.0 A	109.1±9.5 B	92.0±15.8 A	129.6±6.6 C
BMI	27.5±3.4 A	31.3±3.6 AB	27.8±4.9 A	31.9±5.7 B

* Different letters indicate a significant difference between groups to a level of 5%; same letters for different groups indicate no significant difference. TC = Total Cholesterol; TG = Triglycerides; HDL-c = High-Density Lipoprotein; LDL-c = Low-Density Lipoprotein; Castelli Index I = TC/HDL-c; Castelli Index II = LDL-c/HDL-c; WC = Waist Circumference; BMI = Body Mass Index.

Table 2 shows that there were significant differences in the proportion of patients with altered values of HDL-c and non-HDL-c between those with Metabolic Syndrome and those without. 

**Table 2:** Presence of Metabolic Syndrome by normal and altered HDL-c and non-HDL-c levels (%).

**MS**	**HDL-c**		**non-HDL-c**	
	**Normal**	**Altered**	**Normal**	**Altered**
No	43.5%	28.3%	44.9%	21.8%
Yes	56.5%	71.7%	55.1%	78.2%
p-value	0.6767	0.0000	0.3964	0.0000

## DISCUSSION

 In our study, we observed that altered values for both HDL-c and non-HDL-c were related to higher values for glycemia, TC, TG, LDL-c, CI-I, CI-II, WC, and BMI. Presence of MS was also associated with altered values for HDL-c and non-HDL-c. 

 Recently the International Atherosclerosis Society (2015) and the Brazilian dyslipidemia guidelines (2017) have flagged non-HDL-c as the primary form of atherogenic cholesterol and a therapeutic target. 6 ^-^ 8 The guideline recommendation is to use non-HDL-c as a parameter for evaluation of dyslipidemias, especially in patients with higher triglycerides levels (>400mg/dL). This update maintains the recommendation of achieving levels of LDL-c, the primary target, and non-HDL-c, the secondary target, according to cardiovascular risk. The purpose of using non-HDL-c is to estimate the quantity of circulating atherogenic lipoproteins in plasma, especially in subjects with high TG. 6

 Hypercholesterolemia is a clinical condition that may be interrelated with oxidative stress, insulin resistance, hyperglycemia, obesity, and MS. Prognosis includes development of diabetes mellitus, endothelial dysfunction, and atherosclerosis. The relationship between an atherogenic lipid profile and high plasma triglycerides contributes to progression of atherosclerosis. 6 ^-^ 8 For this reason, evaluation of non-HDL-c could play an essential role in the prevention of vascular events. 

 Non–HDL-c is considered a valuable predictor of premature atherosclerosis and coronary events such as myocardial infarction and cardiovascular mortality. 9 It represents the sum of all lipoproteins with atherogenic properties and so, in other words, non-HDL-c consists of atherogenic remnants. 4 ^,^ 6 ^,^ 10 ^,^ 11

 Some studies suggest that aiming to reduce non-HDL-c and disregarding other lipid parameters might be a better approach, because patients with elevated non-HDL-c had a higher risk of cardiac events than those with elevated TG or with high LDL-c. 9 ^,^ 12 ^-^ 15

 Our results show that higher non-HDL-c levels were associated with higher LDL-c levels. Authors have suggested that non-HDL-c levels may reflect the risk of CVD better than LDL-c alone. Furthermore, the connection between atherogenic lipoprotein phenotypes (non-HDL-c) and obesity is more relevant than the connection with LDL-c. 6 ^,^ 9 ^,^ 16

 As can be observed in Table 1 , TG values were significantly higher among those with altered non-HDL-c compared to those with normal non-HDL-c. It is known that the association between non-HDL-c and high TG levels can be described as the co-occurrence of hypertriglyceridemia, low HDL-c, and high levels of small dense-LDL particles. 9 Concomitant increase of plasma TG and apo-B levels accounts for dyslipoproteinemia that is determinant of both DM and MS. 6 ^,^ 9

 The Castelli Risk Indexes I and II can also be used for assessment of cardiovascular risk. 1 There were statistically significant differences in CI-I and CI-II between subjects classified as having normal and altered HDL-c and non-HDL-c ( Table 1 ), indicating that assessment of these indices may be of help in clinical management. To the best of our knowledge, there are no studies involving Castelli indexes and non-HDL-c. 

 Values for non-HDL-c are also closely linked to visceral obesity. 17 Our data showed that non-HDL-c is associated with waist circumference. 

 Studies have shown that patients with MS have higher non-HDL-c levels. 9 ^,^ 15 ^,^ 16 In our study, the proportions of patients with altered HDL-c and non-HDL-c levels were significantly higher among patients with MS than among patients without MS. The mechanisms linking non-HDL-c to MS have not been thoroughly explained, but low-grade inflammation, a pro-coagulatory state, thrombosis, and other cardiovascular complications should all be considered. 

 Studies have shown that high levels of non-HDL-c were associated with higher odds of developing metabolic syndrome, independent of central obesity and insulin resistant states 6 ^,^ 9 ^,^ 14 ^-^ 16 and high non-HDL-c levels are a direct cause of atherosclerosis. This aggression to the vascular endothelium is a risk factor for stroke and cardiovascular morbidity and mortality. 18 ^-^ 20

 Panazzolo et al. 21 showed in non-diabetic women that a multivariate scenario of relations between parameters related to obesity and MS indicates an important association between these pathologies and microvascular reactivity. 

 Kraemer-Aguiar et al. 22 suggested that obesity is sufficient to result in impairment of vascular reactivity, and that BMI may positively correlate with worsening of endothelium changes. 

 In line with the literature, our study shows that elements of the atherogenic lipid profile may assist in achieving clinical goals including prevention and early diagnosis of CVD events. Our results show that both HDL-c and non-HDL-c are associated with insulin resistance, dyslipidemia, atherogenic indices, and obesity. There is therefore a need for randomized clinical intervention trials examining the potential role of non-HDL-c as a possible primary therapeutic target. 
